# Socialistic Theories, in Their Medical Aspect

**Published:** 1856-01

**Authors:** 


### SOCIALISTIC THEORIES IN THEIR MEDICAL ASPECT.

A recent article in the Buffalo *Journal*, by its talented editor, Dr. Hunt, shows the horrid practices of the "Free Love" organization as well as those of the fashionable *isms* of the present day, and, also, that a low and base sensualism is a prominent characteristic of quackery. He refers to certain works published and intimates plainly enough what were, and are, the characters of their authors. Such works as "Love *versus* Marriage," "Passional Hygiene," "Water Cure for Ladies," &c., &c., and that climax of vulgarity and sensuality, "Mary Lyndon," by Mrs. Mary Gove Nichols,—a she-doctor and professor. These and other works are, taken together, an insult to that sentiment of virtue, propriety, and honor characteristic of the pure and refined of either sex.

He shows the sentiments of the founders of some of these *isms* to be loose and immoral, and refers to Miss Catharine E. Beecher's "Letters on Health," for proof of the immoral and shameless conduct of the proprietors of these water cure establishments.

We will publish the entire article in our next issue, that our readers may see what comes out of these "Nazareths," what outrages upon decency are perpetrated, and what abominations are connected with quackery, no matter what may be the aspect it assumes.

The facts should be widely disseminated, that the virtuous and the pure of the gentler sex, who are the especial victims of their vulgar practices, may be saved from the perpetration of these outrages upon their delicacy and modesty.

We have never wearied in our warfare upon the whole army of empirics, indifferent as to their badge or uniform, and we will continue it as well for the moral as the physical benefit of our fellow beings.

